# Extrapleural air secondary to idiopathic pulmonary fibrosis‐related pneumomediastinum

**DOI:** 10.1002/rcr2.1271

**Published:** 2024-01-03

**Authors:** Yuichi Nagata, Takayasu Watanabe, Yuki Tanabe, Motoyasu Kato, Takehito Shukuya, Kuniaki Seyama, Kazuhisa Takahashi

**Affiliations:** ^1^ Department of Respiratory Medicine Juntendo University School of Medicine Tokyo Japan

**Keywords:** coronal reconstruction, extrapleural air, pneumomediastinum, pneumothorax, pulmonary fibrosis

## Abstract

Extrapleural air is a rare condition that may concurrently develop with pneumomediastinum and pneumothorax, especially in older patients with fragile connective tissues. Physicians should consider extrapleural air to prevent inadvertent harm. Coronal reconstruction computed tomography images help appreciate extrapleural air and recognize the track of extrapulmonary air.

## Graphical abstract

Extrapleural air is a rare condition that may concurrently develop with pneumomediastinum and pneumothorax, especially in older patients with fragile connective tissues. Coronal reconstruction computed tomography images help in determining how air from pneumomediastinum is related to other conditions such as extrapleural air and pneumothorax. Extrapleural air is very rare, but it should be differentiated from pneumothorax and closely monitored to prevent inadvertent harm.

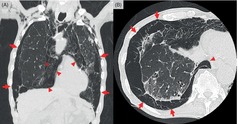

## CLINICAL IMAGE

**FIGURE 1:**
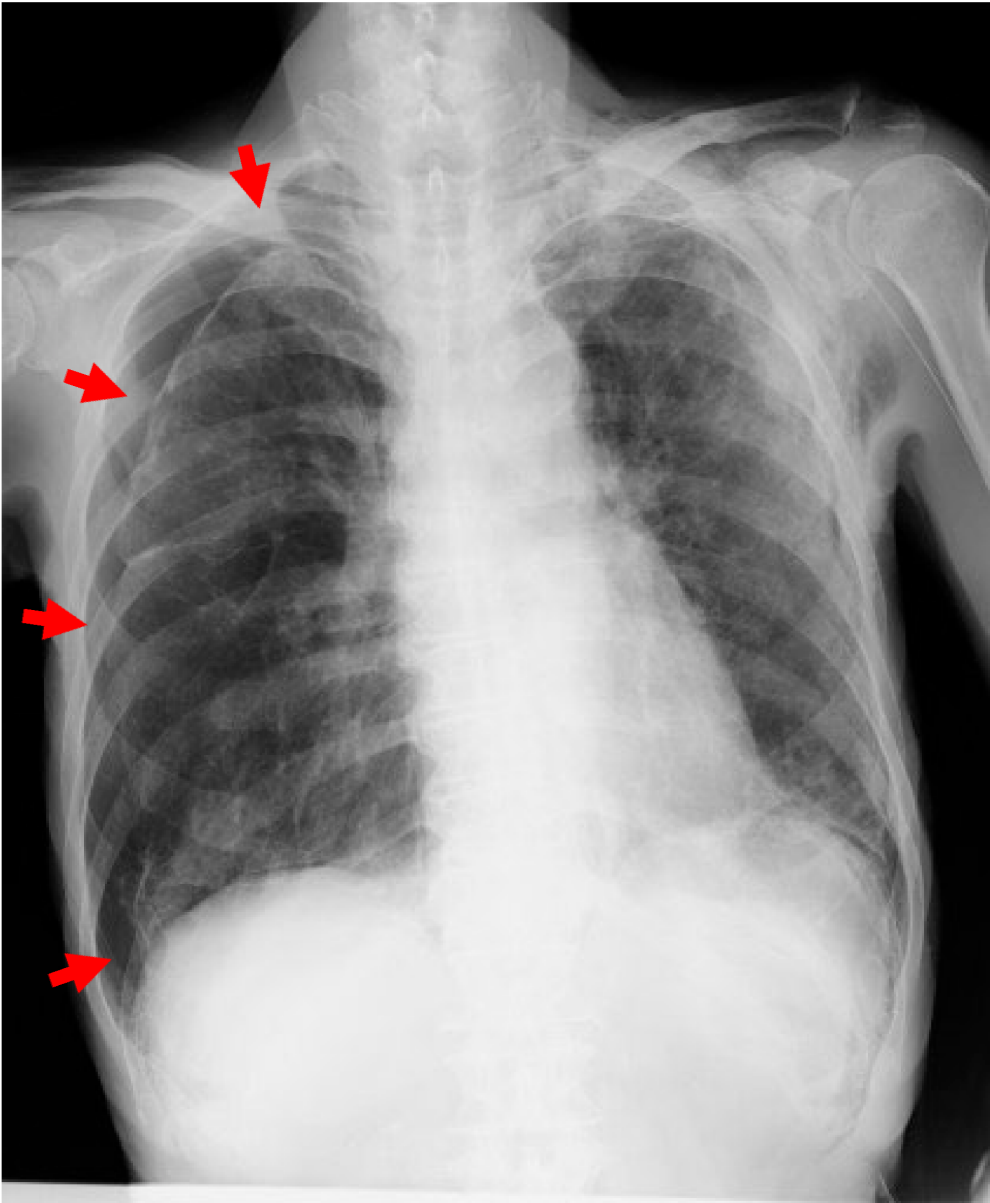
Chest x‐ray image showing the collapse of the right lung from his rib cage.

**FIGURE 2:**
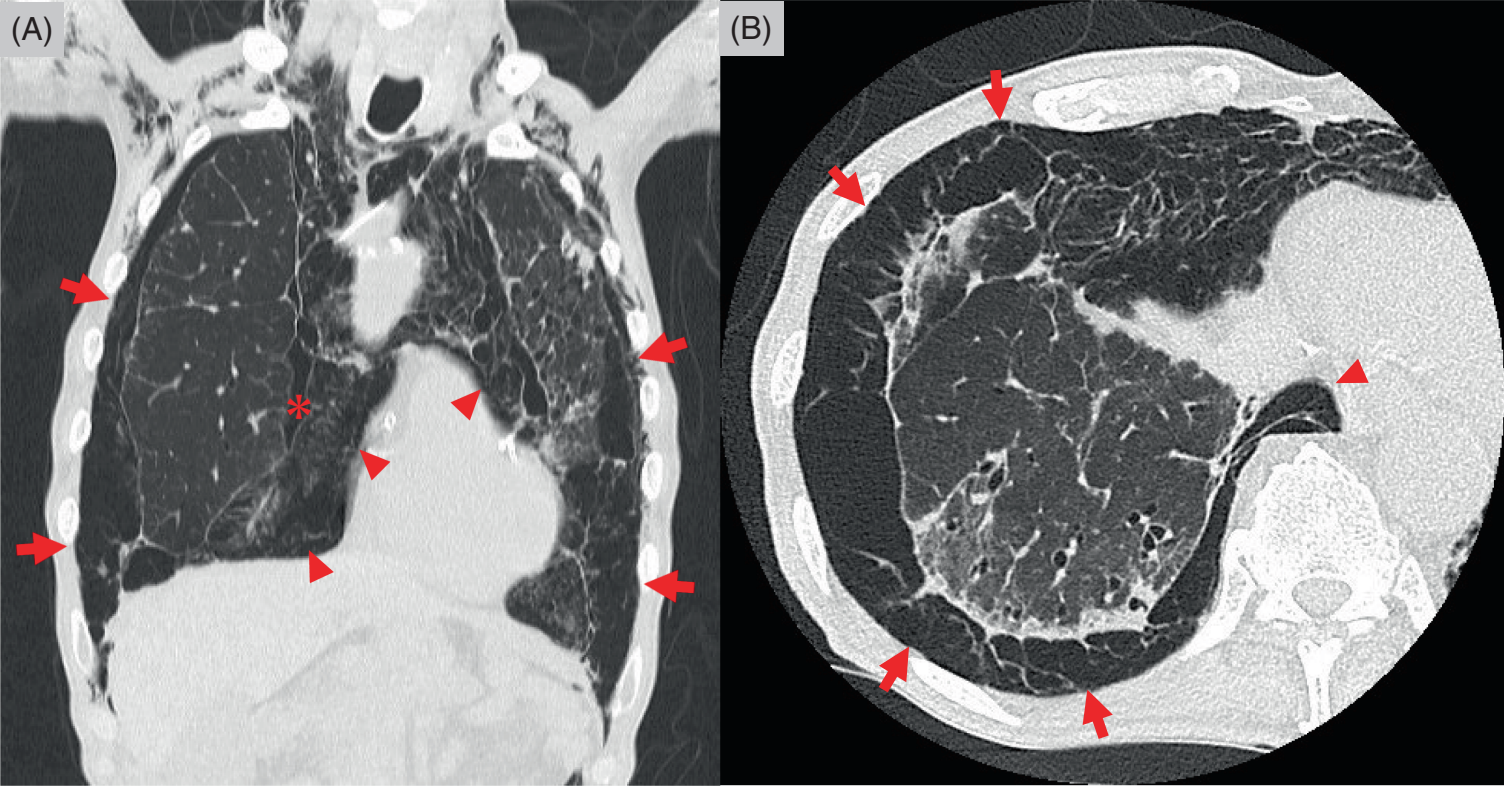
Chest computed tomography scan in coronal (A) and axial (B) views shows a space of fragile connective tissue and mediastinal emphysema surrounding the lungs: extrapleural air.

A 78‐year‐old man with a history of idiopathic pulmonary fibrosis was admitted to our hospital with a small left pneumothorax and mild pneumomediastinum. He was managed conservatively. Two weeks later, a chest x‐ray revealed a lesion mimicking a moderate right pneumothorax, suggesting the need for drainage (Figure 1). Chest computed tomography on the same day showed a space with fragile connective tissue surrounding the lungs (Figure 2, arrows). It was contiguous with mediastinal emphysema (Figure 2, arrowheads). Extrapleural air was diagnosed, and drainage was not performed. Additionally, coronal reconstruction computed tomography images revealed a small air space (Figure 2, asterisk) in the hilar region of the thoracic cavity, indicating pneumothorax. Stretching of the mediastinal pleura by pneumomediastinum can lead to rupture and pneumothorax.^1^ Upon careful observation, extrapleural air and pneumothorax did not exacerbate, and hence, no surgical or drug treatment was administered. He was then transferred to a nursing home.

Coronal reconstruction computed tomography images help in determining how air from pneumomediastinum is related to other conditions such as extrapleural air and pneumothorax. Idiopathic pulmonary fibrosis is characterized by increased elastic recoil force and is commonly observed in older adults with fragile connective tissues due to aging. The air from an alveolar rupture travels along the bronchovascular sheath to the mediastinum and causes pneumomediastinum. Extrapleural air concurrent with pneumothorax may develop. Extrapleural air is very rare, but it should be differentiated from pneumothorax and closely monitored to prevent inadvertent harm.^2^

## AUTHOR CONTRIBUTIONS

Yuichi Nagata, Takayasu Watanabe, Yuki Tanabe, Takehito Shukuya, Kuniaki Seyama, and Kazuhisa Takahashi were the attending physicians who treated the patient on admission. Motoyasu Kato was an outpatient physician. Yuichi Nagata, Motoyasu Kato, and Kuniaki Seyama drafted the manuscript. Yuichi Nagata submitted the final manuscript. All authors have read and approved the final manuscript for submission.

## CONFLICT OF INTEREST STATEMENT

None declared.

## ETHICS STATEMENT

The authors declare that appropriate written informed consent was obtained for the publication of this manuscript and accompanying images.

## Data Availability

Data sharing not applicable to this article as no datasets were generated or analysed during the current study.
